# Clinical and Cytokine Profile of Children With COVID-19: A Report From Turkey

**DOI:** 10.7759/cureus.37139

**Published:** 2023-04-04

**Authors:** Tahir Dalkıran, Emine M Kara, Velid Ünsal, Sadık Yurttutan, Sevcan İpek, Besra Dağoğlu, Yaşar Kandur

**Affiliations:** 1 Department of Pediatric Intensive Care Unit, Kahramanmaraş Necip Fazıl City Hospital, Kahramanmaraş, TUR; 2 Department of Pediatric Infectious Diseases, Istinye University, Istanbul, TUR; 3 Department of Medical Biochemistry, Mardin Artuklu University, Mardin, TUR; 4 Department of Neonatology, Faculty of Medicine, Kahramanmaraş Sütçü İmam University, Kahramanmaraş, TUR; 5 Department of Pediatric Critical Care, Faculty of Medicine, Kahramanmaraş Sütçü İmam University, Kahramanmaraş, TUR; 6 Department of Radiology, Kahramanmaraş Necip Fazıl City Hospital, Kahramanmaraş, TUR; 7 Department of Pediatric Nephrology, Kırıkkale University, Kırıkkale, TUR

**Keywords:** severe cases, pediatric patient, pro-bnp, inflammatory cytokines, covid-19

## Abstract

Background

We aimed to analyze the expression of infection-related biomarkers and inflammatory cytokines in laboratory-confirmed cases and compare the differences between clinically severe and non-severe ones.

Method

We randomly selected 35 patients who were hospitalized with the diagnosis of coronavirus disease 2019 (COVID-19). Blood serum was obtained at the time of admission to the hospital, on the third to the fifth day, and at the time of discharge.

Result

The median age of our patients was 56.5±69.7 months (range: 1-205 months). The mean pro-B-type natriuretic peptide (pro-BNP) was significantly higher at the time of admission than on the third to the fifth day of illness. The mean pro-B-type natriuretic peptide levels at three time points were significantly higher in patients with severe cases than in mild-moderate cases. However, there was no significant difference between the clinical severity with regard to the cytokine levels at disease onset and recovery.

Conclusion

In the study, it was shown that cytokines play an important role in the pathogenesis of COVID-19. Therefore, it may be beneficial to use agents such as tocilizumab in the treatment.

## Introduction

Coronavirus disease 2019 (COVID-19) has become a public health threat to people all over the world, which was declared a pandemic on March 11, 2020. Globally, as reported by the World Health Organization (WHO) on March 21, 2023, there have been 761,071,826 confirmed cases of COVID-19, including 6,879,677 deaths (https://covid19.who.int/). The lower airway tract is the primary target of the infection [1]. Acute respiratory distress syndrome (ARDS), septic shock, and coagulation disorders are severe complications of this infection, which are rare in children [2]. A systemic inflammatory response resulting from the release of large amounts of inflammatory cytokines (interferon (IFN)-α, IFN-g, interleukin (IL)-1β, IL-6, IL-12, IL-18, IL-33, tumor necrosis factor (TNF)-α, TGF-β, etc.) leads to ARDS [3]. The data on the pandemic has shown that children are less susceptible to COVID-19 and seem to be affected less commonly than adults [4,5]. The reason for this can be the difference in severity between adults and children primarily due to the over-activation of the immune system [5]. After all, the difference in severity between adults and children may be related to differences in receptors in the renin-angiotensin system (RAS) and altered inflammatory responses to pathogens [6].

This prospective study aimed to analyze the expression of infection-related biomarkers and inflammatory cytokines (IL-1β, IL-6, and TNF-α) in laboratory-confirmed cases and compare the differences between clinically severe and non-severe ones.

## Materials and methods

We randomly selected 35 patients who were hospitalized with the diagnosis of COVID-19 at the local state and university hospitals during the period between May 25, 2020, and August 12, 2020. Written informed consent was obtained from the parents. Data including demographic characteristics, medical history, symptoms, signs, and laboratory findings were collected from the patients’ medical records. Blood serum was obtained to study routine blood tests, infection-related biomarkers, and inflammatory cytokines (IL-1β, IL-6, and TNF-α) at the time of admission to the hospital, on the third to the fifth day, and at the time of discharge. Serum TNF-α (catalog number MBS267654), IL-1β (catalog number BMS224-2), and IL-6 (catalog number MBS021993) levels were measured using the enzyme-linked immunosorbent assay (ELISA) method. The test results were expressed in pg/mL. The methods were applied in accordance with the protocols provided by the manufacturers of the kits. Measurement was performed using the Multiskan FC photometric microplate reader (Thermo Fisher Scientific, Waltham, MA, USA). The severity of COVID-19 was determined according to the Fifth Revised Trial Version of the Novel Coronavirus Pneumonia Diagnosis and Treatment Guidance [7]. The criteria for severe disease are as follows: respiratory distress with a respiratory rate over 30 per minute, oxygen saturation ≤ 93% in the resting state, and arterial blood oxygen partial pressure (PaO2)/oxygen concentration (FiO2) ≤ 300 mmHg. The ethics committee approval of the study was obtained from the Kahramanmaraş Sütçü İmam University Clinical Research Ethics Committee (date: May 27, 2020; number: 2020/10).

Statistical analysis

We described the categorical variables as frequency rates and percentages, and continuous variables as mean and standard deviation (SD). The T-test and the Mann-Whitney U test were performed for two-group comparisons. All statistical analyses were performed using the Statistical Package for the Social Sciences (SPSS) version 20.0 software (IBM SPSS Statistics, Armonk, NY, USA). Two-sided P-values of less than 0.05 was considered statistically significant. Correlation between laboratory variables and cytokines was sought using a nonparametric test of correlation (Spearman’s rank correlation test).

## Results

The median age of 35 patients in total was 56.5±69.7 months (range: 1-205 months); 18 (51.4%) patients were male. Seventeen (48.6 %) patients had a history of contact with a COVID-19-positive patient. None of the patients received SARS-CoV-2 vaccination. The presenting symptoms were mostly associated with one another, which included fever (n=26, 74.2%), cough (n=20, 57.1%), respiratory distress (n=17, 48.5%), nasal congestion/runny nose (n=9, 25.7%), headache (n=2, 5.7%), myalgia (n=2, 5.7%), and sore throat (n=2, 5.7%). One patient had also abdominal pain. The physical examination findings were as follows: 18 (51.4%) patients had rales/rhonchi on auscultation of the lungs, four (11.4%) patients had cyanosis at hospital admission, 13 (37.1%) patients had wheezing, 10 (28.6%) patients had signs of respiratory disorders (dyspnea, and suprasternal and intercostal retractions), and 16 (45.7%) patients had tachycardia and tachypnea. Two patients were asymptomatic. Ten (28.6%) patients’ clinical presentation was severe, and they were followed in the intensive care unit (ICU). The remaining 23 patients’ clinical severity was mild-moderate. One (2.9%) patient who had a congenital heart defect (transposition of the great arteries (TGA)) died because of the disease. Two (5.7%) patients had cerebral palsy and epilepsy. These patients were intubated and were followed under mechanical ventilation.

The mean leucocyte, neutrophil count, serum pro-B-type natriuretic peptide (pro-BNP), interleukin (IL)-1β, IL-6, and tumor necrosis factor (TNF)-α serum levels were significantly higher at the time of admission than recovery. The mean eosinophil count, mean corpuscular volume (MCV), C-reactive protein (CRP), creatinine kinase (CK), ferritin, aspartate transaminase (AST), and alanine transaminase (ALT) levels were significantly lower at the time of admission. The mean D-dimer, CK level, and ferritin levels were significantly higher at peak disease than at the time of admission. The mean pro-BNP was significantly higher at the time of admission than at the disease peak. Moreover, the pro-BNP level of the patient who died was relatively very high (28,000 pg/mL). Coronavirus PCR was positive in 31 (88.6%) patients at the time of admission, while it became positive at the disease peak in the remaining four (11.4%) patients. After the disease was cured, the PCR became negative in all patients, except the patients who died because of the disease (Table 1).

**Table 1:** Comparison of the laboratory tests and cytokine levels between different time points of the disease course *Comparison between day 3-5 and day 0 SD: standard deviation, CRP: C-reactive protein, pro-BNP: pro-B-type natriuretic peptide, AST: aspartate transaminase, ALT: alanine transaminase, IL: interleukin, TNF: tumor necrosis factor

Variable (reference range)	Day 0 (mean±SD)	Day 3-5 (mean±SD)	Day of discharge (mean±SD)	P (day 0 versus day of discharge)/P (day 3-5 versus day 0)*
Mean leucocyte count/μL	10600±5700	7800±4800	8400±3700	<0.001
Mean neutrophil count/μL	5700±5100	3200±3100	3300±2100	<0.001
Patients with neutrophilia (number (%))/patients with neutropenia (number (%))	5 (14.3)/3 (8.6)	4 (11.4)/7 (20)	2 (5.7)/4 (11.7)	
Mean lymphocyte count/μL	4100±3300	3800±3100	4100±2700	0.889
Patients with lymphocytosis (number (%))	15 (42.9)	8 (22.9)	9 (25.7)	
Patients with lymphopenia (number (%))	2 (5.7)	5 (14.3)	1 (2.9)	
Mean monocyte count/μL	880±390	600±360	627±310	<0.001
Patients with monocytosis (number (%))	6 (17.1)	4 (11.4)	4 (11.4)	
Mean eosinophil count/μL	140±49	200+50	248+59	0.007
Mean hemoglobin (gr/dL)	11.8±1.6	12±1.6	11.8 ±1.6	1.0
Mean platelet count/μL×1000	327±165	334±212	321±130	0.889
Mean CRP (mg/L) (0-5 mg/L)	8.2±4.2	10.0±5.4	11±6.2	0.013
Mean pro-BNP (pg/mL) (70-133 pg/mL)	1630±1100	421±315	452±343	0.001, 0.001*
Mean AST (U/L)	34±25	62±29	38±9	<0.001
Mean ALT (U/L)	21±14	36±16	23±9	<0.001
Mean creatinine (mg/dL)	0.4±0.1	0.4±0.2	0.4±0.2	0.992
Mean albumin (gr/dL)	4.3± 2.0	3.9±1.7	4.0±1.6	<0.001
Mean creatinine kinase (U/L)	160±44	463±312	202±87	0.001 0.001*
Mean ferritin (ng/mL)	126±43	556±470	315±236	0.013 0.001*
Mean D-dimer (ng/mL) (0-500 ng/mL)	387±222	482±596	325±133	0.001, 0.001*
Mean IL-1β (pg/mL) (0.16-10 pg/mL)	30.2±5.7		26.9±5.7	<0.001
Mean IL-6 (pg/mL) (3.12-100 pg/mL)	173.9±26.5		151.9±21.5	<0.001
Mean TNF-α (pg/mL) (15.6-1000 pg/mL)	373.7±44.9		330.2±42.9	<0.001
Patients with coronavirus PCR positivity (number (%))	31 (88)	35 (100)	1 (1)	

Patients with mild-moderate clinical severity were older than those with severe clinical presentation (87.6±15.1 versus 55.1±30.1 months, P=0.304). However, this difference was statistically nonsignificant. The mean neutrophil count and CRP on the third to the fifth day, the mean pro-BNP level at three time points, the mean CK level at the disease peak and the end of the disease, and the mean ferritin and D-dimer levels on the third to the fifth day were significantly higher in patients with severe clinical presentation. However, there was no significant difference between the clinical severity with regard to the cytokine levels at the time of admission and recovery (Table 2).

**Table 2:** Comparison of the laboratory tests and cytokine levels between patients with mild-moderate and severe clinical presentation SD: standard deviation, CRP: C-reactive protein, pro-BNP: pro-B-type natriuretic peptide, AST: aspartate transaminase, ALT: alanine transaminase, CK: creatinine kinase, IL: interleukin, TNF: tumor necrosis factor

Variable	Mild-moderate clinical presentation (N=23) (mean±SD)	Severe clinical presentation (N=10) (mean±SD)	P
Mean age (months)	87.6±15.1	55.1±30.1	0.304
Mean leucocyte count/μL			
Day 0	9700±4700	13000±8000	0.208
Day of discharge	6900±4800	9000±5000	0.353
Mean neutrophil count/μL			
Day 0	5200±800	7800±3200	0.281
Day 3-5	2300±1700	5400±4800	0.028
Mean lymphocyte count/μL			
Day 0	3700±3300	4300±2500	0.708
Day of discharge	3800±3400	2800±1900	0.490
Mean eosinophil count/μL			
Day 0	180±70	54+30	0.307
Day of discharge	235±101	145±75	0.596
Mean hemoglobin (gr/dL)	12.0±1.6	11.9±1.6	0.945
Mean platelet count/μL			
Day 0	308000±100000	406000±282000	0.195
Day of discharge	310000±174000	419000±304000	0.276
Mean CRP (mg/L)			
Day 0	9.8±4.5	4.6±2.4	0.498
Day 3-5	4.2±4.7	25±10	0.007
Day of discharge	2.9±0.2	29.5±21.1	0.083
Mean pro-BNP (pg/mL)			
Day 0	122±63	6100±4500	0.036
Day 3-5	100±33	1900±1800	0.035
Day of discharge	78±20	1480±1280	0.097
Mean AST (U/L)	30±18	46±40	0.174
Mean ALT (U/L)	20±12	26±18	0.377
Mean creatinine (mg/dL)	0.4±0.15	0.4±0.2	0.913
Mean albumin (gr/dL)	4.5±0.5	4.4±0.7	0.925
Mean CK (U/L)			
Day 0	186±64	121±56	0.566
Day 3-5	183±56	1400±1300	0.011
Day of discharge	93±20	490±303	0.05
Mean ferritin (ng/mL)			
Day 0	126±43	556±470	0.534
Day 3-5	82±42	2200±2100	0.045
Day of discharge	82±42	880±820	0.156
Mean D-dimer (ng/mL)			
Day 0	354±179	345±137	0.918
Day 3-5	322±112	933±569	0.05
Day of discharge	294±112	383±172	0.227
Mean IL-1β			
Day 0	29.3±5.7	30.6±6.0	0.636
Day of discharge	26.3±5.3	25.2±5.0	0.645
Mean IL-6			
Day 0	167.9±29.9	177.1±22.1	0.470
Day of discharge	146.9±24	149.7±6.0	0.764
Mean TNF-α			
Day 0	370.7±53.4	378.7±32.7	0.717
Day of discharge	318.8±45.7	341.2±30.0	0.243

Six (17.1%) patients had widespread ground-glass opacity (GGO) and multifocal consolidation appearance on the thoracic computed tomography (CT). Two (5.7%) patients had minimal GGO on CT. Eleven (31.4%) patients took bronchodilators (salbutamol and ipratropium bromide); 33 (91.4%) patients additionally took different antibiotics including azithromycin. None of the patients received tocilizumab. Nine (25%) patients received steroids. Seven (20%) patients took oseltamivir; six (17.1%) patients received Kaletra (lopinavir/ritonavir). Four (11.4%) patients took favipiravir; seven (20%) patients additionally took hydroxychloroquine. Three (8.6%) patients needed nasal continuous positive airway pressure (CPAP) application. Two (5.7%) patients needed mechanical ventilation. One (2.9%) patient died because of COVID-19. The mean duration of COVID-19 PCR positivity was 6.7±2.2 days (Table 3).

**Table 3:** Treatment options ICU: intensive care unit, SD: standard deviation

	Number (%) (N=35)
Number of patients hospitalized in the ICU	15 (42.9)
Number of days of hospitalization in the ICU (mean±SD)	7.6±3.1
Number of patients treated with antibiotics	33 (94.3)
Antiviral	
Oseltamivir	7 (20)
Lopinavir/ritonavir	6 (17.1)
Favipiravir	4 (11.4)
Hydroxychloroquine	7 (20)
Bronchodilators	11 (31.4)
Number of patients in need of oxygen	15 (42.9)
Number of patients in need of steroid use	9 (25)
Mean duration of COVID-19 PCR seropositivity (days) (mean±SD)	10.7±2.2

The correlation analysis showed no correlation between the cytokine levels and blood count parameters, biochemical tests, CRP level, or D-dimer level.

## Discussion

Children constitute about 1% of all COVID-19 cases in Turkey [8]. COVID-19 has a milder course in children than in adults. Previous reports have shown that the proportion of children with high inflammatory markers is low [9]. In the present study, we reported the clinical manifestations and inflammatory markers including cytokines (IL-6, IL-1β, and TNF-α) in 35 Turkish children with PCR-positive COVID-19.

In a pediatric case series from Wuhan, China, the median age of the patients was 6.7 years (range: 1 day to 15 years), which was similar to the figure reported in our study [10]. Our youngest patient was three months old. It is reported that children of all ages can be infected, including newborns [11].

Our short-time experience with COVID-19 showed that symptoms are less severe in children than in adults [12]. Previous reports have indicated that fever and cough are the most common clinical manifestations in children. Our results were consistent with these reports. A considerable number of children in our patient population did not exhibit fever. This means that fever is not a diagnostic sign, especially in children.

In children, the severity increased as the patient’s age decreased. A study reported by Zhen-Dong et al. [13] showed that the proportion of children younger than five years who had a severe clinical presentation was 17.9%. A case study reported a 55-day-old infant with a severe clinical course [14]. Consistently, the mean age of our patients with severe disease was lower than that of the patients with a mild-moderate clinical presentation. We thought that this was due to the fact that the immune system of younger children is not fully developed, and they are more susceptible to infections [15].

Pro-BNP was highest at the time of admission to the hospital. It was also remarkably higher in severe patients and again at the time of admission. This suggests that pro-BNP may be an adverse prognostic factor for COVID-19. Our results are consistent with a previous adult study that showed that NT-pro-BNP might be an independent risk factor for in-hospital death in patients with severe COVID-19. That study enrolled 102 adult patients with severe COVID-19 [16]. Previous studies have also found that NT-pro-BNP is a powerful and independent predictor of mortality in community-acquired pneumonia [17-19].

Our results related to complete blood count parameters are inconsistent with other studies that have shown low proportions of neutrophilia (4.6%), neutropenia (6%), and lymphocytopenia (3%) [20-22]. Our study revealed that 20% of the patients developed neutropenia and 14.3% developed neutrophilia and lymphopenia; these figures were relatively higher than those reported by previous studies. This was caused by our relatively severe patient population.

Chen et al. (2020) [23] found that serum concentrations of IL-6 and TNF-α were high in severe cases in comparison with moderate cases, suggesting that cytokine storms might be associated with disease severity. However, our results are not consistent with that hypothesis. There was no difference in mean cytokine levels between severe and mild-moderate disease groups. This was most probably caused by the timing of blood sampling. We took blood samples at disease onset. If we had taken them at the peak stage of the disease, we might have achieved different results.

One of our patients who was one month old died. She had an underlying disease, namely, the transposition of great arteries (TGA). The COVID-19 test was positive at the onset and peak of the disease. Her pro-BNP was relatively very high at the time of admission. Her clinical course became more severe in hours, so there was no time for taking a CT. However, Guo et al. (2020) [24] showed that patients with underlying congenital cardiac disease are at risk of COVID-19-associated myocardial injury.

According to Lu et al. [10], GGO was seen on CT in 30% of diagnosed children, while local appearance was seen in 18.7% and bilateral patchy appearances in 12.3%. Our results were consistent with those figures.

COVID-19 can be detected in different body specimens. A study with 205 patients showed that nasal swab positivity was 63% and pharyngeal swab positivity was 32% [25]. In our study, COVID-19 PCR positivity was relatively high at the time of admission to the hospital. We believe that the effective use of swab sampling is more complicated in adults than in children. Also, our patient population was composed mostly of in-hospital patients compared to the patients in the abovementioned study. Hence, taking specimens may be sloppy in outpatients. Therefore, there may be false-negative results in adult patients. An alternative diagnostic test is the genetic sequencing of specimens.

Most reports have suggested supportive treatment, including oxygen therapy and antibiotics for bacterial superinfections [26]. Antivirals should be used in severe cases; however, there are doubts about their efficacy in COVID-19 cases [27]. We used oseltamivir empirically in our patients since we were in the influenza season during the study period. In our patients, antibiotics were more effective than antivirals and hydroxychloroquine; this means that superinfections may play a role in the COVID-19 pandemic. Azithromycin was a preferred option since the guide of our National Health Commission suggested its usage during the pandemic [28]. Treatment regimens were applied to our patients according to the guidelines of that time [29]. During the disease period, hydroxychloroquine, oseltamivir, and azithromycin were included in the recommendations of the World Health Organization, and we applied these treatments to our patients. However, among the treatment modalities, these drugs are no longer among the treatment recommendations because of their side effects or because of their ineffectiveness [30]. Especially, patients in the ICU benefited from antibiotics, which showed that bacterial superinfections play an important role in the progress of COVID-19. The WHO and CDC have recommended that systemic glucocorticoids should be used in special COVID-19 cases, such as those with exacerbation of chronic obstructive pulmonary disease. The duration of PCR positivity in our population was the same as in the previous reports.

Our study has some limitations. First, we did not evaluate the cytokine levels at peak disease. Second, the patient group is not homogenous since there are more severe patients than those included in previous studies. Third, this study only included a small number of patients; thus, the results should be interpreted with caution, and statistical nonsignificance may not rule out differences between severe and moderate cases.

## Conclusions

In conclusion, our pediatric experience with COVID-19 showed that mortality is relatively low even in the ICU compared to adults. Moreover, cytokines play an important role in the pathogenesis of COVID-19. So, specific treatment options such as tocilizumab (anti-IL-6) may be utilized for clinically critical patients. Cytokines are often elevated in children with COVID-19, but we did not note any association of cytokine levels with disease severity.
